# Key Markers and Epigenetic Modifications of Dental-Derived Mesenchymal Stromal Cells

**DOI:** 10.1155/2021/5521715

**Published:** 2021-05-08

**Authors:** Jingqiu Chen, Xiaodan Zheng, Nanquan Rao, Yao Huang, Juan Liu, Yanhong Li, Jun Zhang

**Affiliations:** Yunnan Provincial Key Laboratory of Stomatology, The Affiliated Stomatology Hospital of Kunming Medical University, Kunming, Yunnan, China

## Abstract

As a novel research hotspot in tissue regeneration, dental-derived mesenchymal stromal cells (MSCs) are famous for their accessibility, multipotent differentiation ability, and high proliferation. However, cellular heterogeneity is a major obstacle to the clinical application of dental-derived MSCs. Here, we reviewed the heterogeneity of dental-derived MSCs firstly and then discussed the key markers and epigenetic modifications related to the proliferation, differentiation, immunomodulation, and aging of dental-derived MSCs. These messages help to control the composition and function of dental-derived MSCs and thus accelerate the translation of cell therapy into clinical practice.

## 1. Introduction

Mesenchymal stromal cells (MSCs) are plastic-adherent fibroblast-like cells [1] that have self-renewal and multidifferentiation potential and strong proliferative ability [2–4]. Bone marrow mesenchymal stromal cells (BMMSCs) are widely recognized in tissue regeneration. However, there are several challenges to obtaining BMMSCs, including pain, morbidity, and low harvested cell number. Thus, alternative sources for BMMSCs must be identified [5, 6]. Dental-derived MSCs have unique clinical advantages, such as easy access and remarkable tissue reparative/regenerative potential, and they have been proposed as ideal candidates for MSCs-based tissue regeneration [7–9].

Tissue regeneration and maintenance are dependent on MSCs [10]. However, a barrier to realize the therapeutic potential of MSCs is their intrinsic heterogeneity, which is also observed for dental-derived MSCs [11–13]. Cell subpopulations within heterogeneous MSCs cultures vary in their regenerative potential, including proliferation potential [14], differentiation [15], and immunomodulatory ability [16]. In MSCs therapy, cells are the active substances in medicines. Although variation is inevitable, our limited ability to detect and control heterogeneity poses challenges for the production of MSCs therapies. Heterogeneity has been cited as a possible factor contributing to the variability in treatment outcomes of MSCs therapies in clinical trials [17–19]. Variation in the regenerative potential among cell subsets in MSCs cultures may confound trial results and slow or arrest the translation of MSCs therapy into clinical practice [20].

Cell-to-cell variation in MSCs function is initiated in vivo in the MSCs niche [21], is evident within single-cell-derived MSCs colonies, and is exacerbated by replicative stress during in vitro cultivation [22]. A focus of ongoing research on dental-derived MSCs heterogeneity is to elucidate key markers. Cellular key markers can be used to noninvasively and nondestructively isolate specific cell subpopulations from MSCs cultures for clinical applications and research [23]. It may also help to regulate the regenerative potential of dental-derived MSCs. Besides, recent studies suggest that epigenetic alterations in cells occur in response to intrinsic cellular inheritance and external environmental stimuli to maintain the homeostasis of cells and niche cells [24, 25]. Epigenetic modification stimulates potentially reversible changes to gene expression, thus presenting exciting opportunities for clinical dental-derived MSCs interventions. Here, we first reviewed the heterogeneity of dental-derived MSCs. Then, we discussed the key markers and epigenetic modifications that support regeneration potential.

## 2. Dental-Derived MSCs and Their Heterogeneity

The oral region contains a variety of distinct MSCs populations, including mesenchymal stromal cells of dental pulp (DPSCs) [26], mesenchymal stromal cells of apical papilla (SCAPs) [27], mesenchymal stromal cells of human exfoliated deciduous teeth (SHEDs) [28], mesenchymal stromal cells of periodontal ligament (PDLSCs) [29], dental follicle cells (DFCs) [30], mesenchymal stromal cells of gingiva (GMSCs) [31], and mesenchymal stromal cells of human tooth germ (TGSCs) [32]. These dental-derived MSCs have less cytoplasm and are spindle-shaped, and they are positive for CD13, CD29, CD44, CD73, CD90, CD105, CD106, CD146, CD166, and CD271 but negative for CD3, CD8, CD11b (or CD14), CD15, CD19 (or CD79*α*), CD33, CD34, CD45, CD71, CD117, and HLA-DR [26, 27, 29, 33–35]. Since oral tissues develop from migrating cranial cells, dental-derived MSCs display ready availability and high proliferation ability [36, 37]. Moreover, they possess multidifferentiation potential and can differentiate into adipocytes [32, 38], chondrocytes [39, 40], osteoblasts [41, 42], neuronal cells [43–45], and endothelial cells [46, 47] (Table 1).

Issues related to cell heterogeneity are getting more and more attention in the field of MSCs research. A more comprehensive understanding of the variability of transplantable populations will help maximize the potential of any MSCs therapy [48]. According to the International Society for Cellular Therapy, dental-derived MSCs meet all the minimum criteria that define MSCs [49]. Even so, this definition does not necessarily identify a homogeneous population of MSCs. Instead, it describes a group of heterogeneous cells that exhibit variability at the level among tissues of origin, individual donors, clonal subpopulations, and single cells [12, 50, 51].

### 2.1. Tissue and Donor Heterogeneity and Functional Variation

Every tissue or organ has its evolutionary origin. The composition of a tissue, in terms of cell types, stands behind the overall functionality. The heterogeneity of cell types increases throughout the evolution in every functional entity [52]. A large amount of evidence shows that MSCs from different tissues have differences in marker profiles, gene expression patterns, and tendency to differentiate into specific cell types [38, 53–55]. GMSCs were isolated and characterized as 90% derived from neural crest cells (cranial neural crest cell-derived GMSCs, N-GMSCs) and 10% derived from the mesoderm (mesoderm GMSCs, M-GMSCs). N-GMSCs express a high level of Fas-ligand (FasL), which induces T cell apoptosis and immune tolerance [56]. In comparison with M-GMSCs, N-GMSCs are more likely to differentiate into chondrocytes and neural cells in vitro and induce T-cell apoptosis [57]. Moreover, N-GMSCs can be induced into neural crest stem-like cells via the activation of RhoA-ROCK/Yes-associated protein 1 (YAP1) signaling [58]. These results indicate that N-GMSCs possess a superior capacity for immune regulation and differentiation than M-GMSCs. Indeed, there is mounting evidence that cultured cells retain a “memory” of their previous environments [59–61]. The highly heterogeneous nature of DPSCs is believed to be due to multiple progenitor cell populations existing in different locations of the dental pulp that may have different proliferation and differentiation abilities [62]. Different niches have been found in situ associated with the vasculature, within the pulpal stroma, in the subodontoblast layer, and among peripheral nerve-associated glial cells [63].

In addition, even from the same tissue source, dental-derived MSCs show tremendous variability between donors [13, 64, 65]. Similarly, as donor age increases, MSCs availability, self-renewal capacity, and differentiation potential have been reported to decline. Many animal and human studies have established the effects of increasing donor chronological age on the impairment of dental-derived MSCs regenerative capabilities [66–69]. In studies that involved small numbers of clones obtained from multiple donors, differences in gene expression among clones obtained from multiple donors might have reflected the different genetic backgrounds of the donors, rather than phenotypic differences between multipotent MSCs and committed progenitor cells [70]. Analysis of gene expression profiles among clones obtained from a single donor may allow researchers to eliminate the differences in genetic backgrounds that are associated with the use of multiple donors [71].

### 2.2. Clonal Heterogeneity and Functional Variation

Further study revealed that the clonal variation of dental-derived MSCs superimposes the difference among tissue- and donor-dependent differences. For example, DPSCs from multiple colonies can reach more than 120 population doublings (PDs), while single-colony-derived DPSCs can proliferate only up to 10-20 PDs [13]. Approximately two-thirds of DPSCs derived from single colonies can form the same amount of dentin as multicolony DPSCs. The other one-third generated only a limited amount of dentin. Studies have demonstrated that each colony is originally derived from the clonal expansion of a single progenitor cell [72]. However, there is growing appreciation that cellular phenotype can be highly variable, even within a clone [22]. Within the same colony, DPSCs of different cell shapes and sizes may be observed. If seeded on dentin, some DPSCs will be transformed into odontoblast-like cells with polarized cell bodies and a cell process extending into the existing dentinal tubules [73, 74]. When SHEDs clones derived from a single colony are transplanted into immunodeficient mice, only a quarter of the clones have the same potential to generate ectopic dentin-like tissue equivalent to that produced by multicolony-derived SHEDs clones [28]. Thus, cell-to-cell variation exists at every level, where the heterogeneity between clones has been noticed, and this must be taken into account when using this cell type in any basic scientific research or clinical application [22].

Functional variation at multiple levels extends to the molecular and epigenetic status of MSCs [71, 75]. Young et al. reported the ability of murine DPSCs clones derived from single cells to differentiate into immature neuron-like cells and oligodendrocyte-like cells in vitro. Significantly, only DPSCs clones with high nestin mRNA expression levels successfully differentiated into microtubule-associated protein 2 (Map2) and neurofilament- (NF-) positive neuron-like cells [50]. Alraies et al. identified differences between high (A3) and low (A1 and A2) proliferative capacity DPSCs populations, even from the same pulpal tissue sample [76]. They found that high proliferative capacity DPSCs exhibited longer telomeres but lacked CD271. It suggested that proliferative and regenerative heterogeneity is related to contrasting telomere lengths and CD271 expression between DPSCs populations. A highly dynamic histone modification response was evident in mineralizing DFCs, but not in DPSCs, and the latter cell type expressed higher levels of the pluripotency-associated genes octamer-binding transcription factor- (OCT-) 4 and NANOG [77]. The authors concluded that the two neural crest-derived MSCs populations were distinguished by epigenetic repression of dentinogenic genes and the dynamic histone enrichment in DFCs during mineralization. It highlighted the essential role of epigenetic mechanisms in the terminal differentiation of dental-derived MSCs and lineage commitment.

Such functional variability may provide an opportunity to identify MSCs subpopulations that are most suitable to drive a series of tissue restoration [75]. Moreover, it motivates ongoing work to reveal molecular or epigenetic markers of MSCs differentiation potential, as discussed later in this review.

## 3. Key Markers of Dental-Derived MSCs

For the application of dental-derived MSCs in tissue engineering and regenerative medicine, it is important to optimize their isolation and preserve their phenotypic properties. The presence of key markers in the MSCs niche will help to break down the heterogeneous barrier of dental-derived MSCs [20, 78]. This section summarizes the key molecules that regulate dental-derived MSCs proliferation, differentiation, immunomodulation, and aging in the MSCs niche. This study will provide key targets and a certain theoretical basis for maintaining MSCs characteristics and promoting MSCs-mediated tissue regeneration.  Table 2 lists some key markers in the field of dental-derived MSCs.

### 3.1. Key Markers of Proliferation Potential

Dental-derived MSCs are a reliable cell resource for tissue regeneration, and they need to be expanded largely in vitro, which requires cells to have superior proliferation and self-renewal potential [79]. It is necessary to explore the key markers related to proliferation to provide useful information for obtaining high-quality MSCs.

STRO-1 is a marker that recognizes a trypsin-insensitive epitope on perivascular cells, and it has been used to isolate MSCs populations from human and rat dental pulp and has shown enhanced proliferation potential [80]. Ranga Rao et al. found a gradual decrease in STRO-1 and transcription factor expression (OCT4, NANOG, and nestin) with an increase in the passage number of GMSCs [14]. A previous study showed that the STRO-1^+^/CD146^+^ SCAPs demonstrated higher colony-forming unit (CFU) efficiency and much higher expression of several embryonic and neural markers (stage-specific embryonic antigen-3 (SSEA-3); Nanog; OCT3/4; and nestin) than nonsorted SCAPs and the STRO-1^−^/CD146^+^ subpopulation [81]. Moreover, STRO-1^+^ selected DPSCs show effective hard tissue formation when seeded into a calcium phosphate ceramic scaffold [82, 83]. These results suggested that STRO-1^+^ cells may represent a very promising adult MSCs source with enhanced multipotent MSCs properties.

Alkaline phosphatase (ALP) is abundantly expressed in undifferentiated cells, such as induced pluripotent stromal (iPS) cells/embryonic stromal (ES) cells, preimplantation embryos (2-cell embryos to blastocysts (inner cell mass)) and embryonic ectoderm at the egg-cylinder stage, primordial germ cells (PGCs), and immature spermatogenic cells [84]. ALP is also a marker of neuronal progenitor cells, human myogenic progenitor cells (also called “pericytes”), and BMMSCs [85–87]. Inada et al. found that among the five primarily isolated SHEDs, two exhibited higher degrees of ALP activity and higher OCT-3/4 expression. Furthermore, these two lines proliferated faster than the other three lines and were easier to program into iPS cells [88]. Moreover, Yu et al. found that the ALP^+^ subpopulation of PDLSCs had higher levels of STRO-1 and CD146 than ALP^−^ cells, even after a high number of passages. ALP^+^ cells expressed significantly higher levels of stemness-associated genes, NANOG, OCT-4, and sex-determining region Y-box- (SOX-) 2 than ALP^−^ cells [89]. In summary, ALP^+^ cells may represent a population with a higher proliferation rate than ALP^−^ cells. Further studies are needed to understand the roles of ALP in stemness in other dental subpopulations.

Nuclear factor I-C (NFIC) is regarded as a key regulator of tooth development. NFIC deficiency causes aberrant odontoblasts and abnormal dentin and periodontium formation, and it ultimately leads to short molar roots [90]. Zhang et al. found that overexpression of NFIC increases cell proliferation in SCAPs [91]. NFIC silencing could prolong the G1 phase of the cell cycle in SCAPs [92]. Moreover, Zhang et al. demonstrated that NFIC can markedly promote the proliferation of rat DFCs [93].

### 3.2. Key Markers of Differentiation Potential

The multipotent properties of dental-derived MSCs make them a valuable cell source for regeneration [6]. Osteogenic differentiation, chondrogenic differentiation, and adipogenic differentiation are the minimum requirements for the differentiation ability of MSCs. In addition, vascularity and innervation are two properties that cannot be sacrificed when considering tissue regeneration. In particular, a limitation of the apical foramen is that it requires the ingrowth of nerve fibers and blood vessels from apical tissues when regenerating parts of the tooth [94].

CD146 is a cell adhesion molecule and an integral membrane glycoprotein at the intercellular junction. It was originally identified as a tumor marker for melanoma [95]. Additionally, CD146 is a MSCs marker that is associated with angiogenic, neurogenic, and mineralization abilities [96]. CD146^+^ BMMSCs possess high migration ability and are stromal cells that support hematopoiesis [97]. Matsui et al. found that CD146^+^ DPSCs have higher mineralization ability than nonseparated cells, CD146^−^ cells, and CD146^+/-^ cells. Moreover, transplanted CD146^+^ cells generated clear dentin/pulp-like structures in immunocompromised beige mice. Immunohistochemical studies detected dentin matrix protein-1 (DMP1), dentin sialophosphoprotein (DSPP), and human mitochondria in transplanted DPSCs [98]. This result suggests that CD146^+^ cells may exhibit a high osteoblastic potential, which is consistent with previous studies [99].

CD271 or p75 neurotrophin receptor (NTR) is a well-conserved transmembrane pro-neurotrophin/neurotrophin receptor that plays critical roles in the maintenance of nerve cell viability [100]. CD271 has been proposed to be a neural MSCs marker that defines a cell population with neurogenic potential in the adult brain subventricular zone (SVZ) [101] and subgranular zone (SGZ) [102]. CD271 is expressed at low levels (<10%) in DPSCs. CD271^+^ DPSCs have higher expression levels of SOX1 (neural precursor cell marker), SOX2 (cell pluripotency marker), and nestin (neural stem cell marker) than CD271^−^ DPSCs. This result suggests that CD271^+^ DPSCs may denote a subpopulation with greater neurogenic potential [103]. In addition, Alvarez et al. used a combination of the three surface markers CD51/CD140*α*, CD271, and STRO-1/CD146 to isolate homogenous populations of PDLSCs. CD271^+^ cells had a higher dental/osteogenic potential and led to the greatest upregulation of osteogenic marker genes, like distal-less homeobox 5 (DLX5), runt-related transcription factor 2 (RUNX2), and bone gamma-carboxyglutamate protein (BGLAP) during the induction process [15].

CD34 is a transmembrane phosphoglycoprotein that was discovered for the first time in hematopoietic stromal cells (SCs). Clinically, it is related to the selection and enrichment of hematopoietic SCs during bone marrow transplantation [104, 105]. In addition, CD34 is assumed to act as a negative marker for MSCs [49]. Pisciotta et al. found that STRO-1^+^/c-Kit^+^/CD34^+^ DPSCs showed a much higher efficiency of commitment compared to STRO-1^+^/c-Kit^+^/CD34^−^ DPSCs, which was demonstrated by the expression of *β*-III tubulin and the shift to a neuron-like shape following the induction [106]. Moreover, Carnevale et al. demonstrated that STRO-1^+^/c-Kit^+^/CD34^+^ DPSCs expressed Schwann cell markers, such as p75NTR, glial fibrillary acidic protein (GFAP), and S100 calcium binding protein B (S100B), after incubation in appropriate induction media. The integration of the graft of the DPSCs-collagen scaffold complex into a sciatic nerve defect in rats contributed directly to nerve fiber regeneration and myelination in vivo [107].

Wnt inhibitory factor 1 (WIF1) belongs to a family of secreted modulators of Wnt proteins. A recent study suggested that WIF1 may enhance the dentinogenic differentiation potential in SCAPs via its regulation of OSX. Moreover, in vivo transplantation experiments revealed that dentinogenesis in SCAPs was enhanced by WIF1 overexpression [108]. Other members of the Wnt modulator family, including secreted frizzled-related proteins (sFRPs), play different roles in Wnt signaling depending on the cell subtype and model used [109–112]. Guanine nucleotide binding proteins (GNAIs) are a family of regulatory proteins responsible for molecular signal transduction of extracellular signals to the intracellular environment [113]. GNAI3 has been demonstrated to play a role in regulating various cellular processes, including proliferation, cytokinesis, apoptosis, migration, and invasion [114–116]. GNAI3 is primarily expressed in Hertwig's epithelial root sheath (HERS) and the surrounding mesenchyme in mice. Moreover, knockdown of GNAI3 could inhibit the proliferation, migration, and odonto/osteogenic differentiation of CD90^+^/CD44^+^/CD45^−^/CD14^−^ SCAPs by inactivating c-Jun N-terminal kinase (JNK) and extracellular-signal regulated kinase (ERK) signaling pathways [117].

### 3.3. Immunomodulatory Key Markers

MSCs-based immunomodulation may play an essential role in the regeneration of different tissues. The immunomodulatory and tropic capacity of transplanted MSCs contributes to the creation of a microenvironment that promotes the activation of endogenous tissue repair mechanisms, and it is now considered to be the major mechanism underlying the therapeutic effects of these cells in vivo [118]. Similar to MSCs from other tissues, dental-derived MSCs possess a strong immunomodulatory ability [6, 119, 120]. Potential mechanisms underlying the immunomodulatory effects of MSCs include enzyme expression, soluble factor production, and cell-to-cell contact [121].

STRO-1^+^ cells in MSCs have significantly enhanced inhibitory effects on lymphocyte proliferation compared with STRO-1^−^ cells; thus, STRO-1^+^ cells impart stronger immunoregulatory effects than STRO-1^−^ cells [122, 123]. A previous study showed that the STRO-1^+^ CD146^+^ subpopulation of PDLSCs inhibit T cell proliferation by suppressing the expression of the nonclassical major histocompatibility complex-like glycoprotein CD1b on dendritic cells [124]. The priming of dental-derived MSCs with interferon-gamma (IFN-*γ*), tumor necrosis factor- (TNF-) *α*, and interleukin- (IL-) 1*β* usually enhances their immunosuppressive ability and could be considered a feedback mechanism that dampens exacerbated immune responses [121]. A recent study of human DPSCs showed that their ability to inhibit peripheral blood mononuclear cell (PBMC) proliferation and B cell immunoglobulin production was significantly enhanced by IFN-*γ* and inhibited by anti-IFN-*γ* antibodies [125].

### 3.4. Key Markers of Cellular Aging

MSCs aging is a negative process from the perspective of cell-based therapies because all advantageous functions may become limited with age. Dental-derived MSCs show clear losses in proliferation capacity with increasing donor age, and they also show donor age-related decreases in maximal life span and proliferation rate [126]. Under standard cell culture conditions, DFCs exhibit cellular senescence after being expanded by more than 14 cell passages [127]. With aging, the proliferation and osteogenic/adipogenic/chondrogenic differentiation potential of PDLSCs decreased while the apoptosis of PDLSCs increased. Moreover, the immunosuppressive ability of PDLSCs decreased with aging [128].

Signs of senescent cells include cell growth arrest, DNA damage foci, and senescence-associated *β*-galactosidase expression, and identifying these markers represents a reliable method for detecting senescent cells [129]. Horibe et al. isolated DPSCs subsets based on their migratory response to granulocyte colony stimulating factor (G-CSF) (MDPSCs) from young and aged donors. In long-term culture, MDPSCs showed a small age-dependent increase in senescence-associated *β*-galactosidase (SA-*β*-gal) production and senescence markers, including p16, p21, IL-1*β*, IL-6, IL-8, and Gro*α*. The regenerative potential of aged MDPSCs was similar to that of young MDPSCs in an ischemic hindlimb model and an ectopic tooth root model [130]. Autologous transplantation of MDPSCs with G-CSF in pulp-ectomized teeth in dogs augmented the regeneration of pulp tissue. Furthermore, MDPSCs from aged donors were as potent as those from young donors [131]. Notably, MDPSCs showed no significant age-related changes in biological properties, such as stability, regenerative potential, and senescence marker expression.

The Hippo pathway is a newly discovered signaling network that is evolutionarily and functionally conserved and has been shown to play a critical role in controlling organ size by regulating both cell proliferation and apoptosis [132, 133]. As a Hippo signaling transcriptional coactivator, YAP plays pivotal roles in MSCs fate and organ size control [134]. Jia et al. discovered that activated YAP promotes proliferation, accelerates the cell cycle, inhibits apoptosis, and delays senescence in human PDLSCs [135]. Knockdown of YAP inhibits the proliferation activity and induces apoptosis of human PDLSCs with the involvement of the Hippo pathway and shows crosstalk with the Erk and Bcl-2 signaling pathways [136]. Transforming growth factor- (TGF-) *β*1 is a potent stimulator of tissue regeneration and is abundant in the bone matrix [137]. Salkin et al. found that TGF-*β*1 transfection has a positive effect on proliferation and the cell cycle and prevents cellular senescence and apoptosis. They suggested that TGF-*β*1 overexpression with gene transfer may improve the biological potential of DPSCs and could represent an option instead of transmission of recombinant protein into cells from the outside [40]. However, the effects of TGF-*β*1 associated with cell senescence are controversial. A previous study demonstrated that treatment with TGF-*β*1 induced PDLSCs senescence, which is characterized by increasing in senescence-associated *β*-galactosidase activity and both p16 and p21 expression. Furthermore, TGF-*β*1 treatment demonstrated the capacity to induce the production of reactive oxygen species (ROS). Of note, the addition of a ROS scavenger successfully rescued TGF-*β*1-induced PDLSCs senescence [138]. These results indicated that the regulatory mechanism of TGF-*β*1 in cell senescence is quite different in various cells.

## 4. Epigenetic Modifications in Dental-Derived MSCs

Epigenetic modifications regulate gene expression without changing the DNA sequence and affect cell development and differentiation. DNA methylation, histone posttranslational modification, and noncoding RNA play primary roles in epigenetic mechanisms [139]. The rescuing potential of MSCs is under the control of different kinds of signals, including the environment, which epigenetically regulate their differentiation processes [140]. Recently, the epigenetic modifications that regulate dental-derived MSCs were revealed; thus, the use of epigenetics to improve the therapeutic potential of dental-derived MSCs has been highlighted. Therefore, summarizing these multiple epigenetic modifications associated with the differentiation process and determining how these modifications could be reversed are of paramount importance.  Table 3 lists some epigenetic biological targets in the field of dental-derived MSCs.

### 4.1. DNA Methylation

DNA methylation is an essential epigenetic mechanism that plays a vital role in the development and differentiation of early embryos by regulating gene expression patterns. The global DNA methylation landscapes of early-life human tissues, such as oocytes, blastocysts, or placenta, are characterized by specific genome-wide hypomethylation compared to differentiated tissue postimplantation [141]. A previous study found that SHEDs have partially methylated domains (PMDs) that are close to the inner cell mass (ICM) and placental methylome. The methylation status of related genes changes under inflammation. For example, 44% of normal dental pulp tissues show complete methylation, while 93% of inflamed dental pulp tissue samples contain IFN-*γ* genes that are only partially methylated or unmethylated. In addition, IFN-*γ* transcription does not occur in the pulp tissue that shows per-methylation [142].

DNA methylation refers to the process in which methyl groups are transferred to cytosine bases of DNA and converted into 5-methylcytosine [143]. This process is catalyzed by DNA methyltransferases (DNMTs). The DNA methyltransferase family includes DNMT1, DNMT2, DNMT3a, DNMT3b, and DNMT3L. Several studies suggest that DNA demethylation levels are correlated with the osteogenesis capacity of MSCs and that DNMT inhibitors could downregulate DNA methylation to improve osteogenesis [144, 145]. 5-Azacytidine (5-aza), a DNMT inhibitor, works by integrating into the DNA structure to prevent DNA from interacting with DNMTs, and it also stimulates DNMT degradation [146]. Liu et al. found that high glucose conditions increased the DNA methylation levels of PDLSCs and blocked osteogenic differentiation ability. 5-Aza-2′-deoxycytidine (5-aza-dC) could rescue the osteogenic differentiation capacity of PDLSCs through activation of the canonical Wnt signaling pathway and the upregulation of osteogenesis-related genes (ALP, OCN, osteopontin (OPN), and OSX) [147]. Upon treatment with 5-aza-2′-deoxycytidine (5-aza-CdR), the odontogenic differentiation capacity of DPSCs is enhanced. 5-Aza-CdR upregulates odontogenic markers (DSPP and DMP1) and transcription factors (RUNX2, DLX5, and OSX), increases ALP activity, and accelerates calcified nodule formation [148]. In addition, myogenic differentiation is also improved after treatment with 5-aza. Nakatsuka et al. used 5-aza to investigate the myogenic differentiation potential of mouse DPSCs. DNA demethylation induced by 5-aza and forced expression of myogenic differentiation 1 (Myod1) upregulated muscle-specific transcription factors, such as myogenin and paired box 7 (Pax7) [149].

The ten-eleven translocation (Tet) family is a group of recently identified demethylases capable of modifying DNA by hydroxylating 5-methylcytosine (5-mC) to 5-hydroxymethylcytosine (5-hmC) [150]. Three Tet family members (Tet1, Tet2, and Tet3) show distinct expression patterns depending on the cell or tissue type and developmental stage [151, 152]. This discovery revealed a new mechanism by which the Tet enzyme regulates DNA demethylation. Yu et al. found that downregulation of Tet1 and Tet2 led to the hypermethylation of the Dickkopf WNT signaling pathway inhibitor 1 (DKK-1) promoter, activated the WNT signaling pathway, and increased the expression of FasL, and it also improved the immune regulation ability of PDLSCs. Importantly, Tet1/Tet2-downregulated PDLSCs showed a significantly increased therapeutic effect on DSS-induced colitis mice [153]. This result indicated that the Tet/DKK-1/FasL cascade may serve as a promising target for enhancing PDLSCs-based immune therapy.

### 4.2. Histone Posttranslational Modifications

The posttranslational modification of histones mainly occurs at the N-end of the tail protruding from the nucleosome core, and this modification plays an essential role in chromatin remodeling and gene expression regulation. In detail, distinct histone amino-terminal modifications can generate synergistic or antagonistic interaction affinity for chromatin-associated proteins, which in turn dictate dynamic transitions between transcriptionally active or transcriptionally silent chromatin states [154]. The more common histone modifications include methylation, acetylation, phosphorylation, and ubiquitination. Among them, histone acetylation has been widely studied in the field of dental-derived MSCs [155]. Acetylation is the only modification that directly causes a structural relaxation of chromatin by neutralizing the charge of histones [156]. The balance of the acetylation process depends on the role of histone acetyltransferases (HATs) and histone deacetylases (HDACs). HATs cause histidine acetyltransferases to add negatively charged acetyl groups, which weaken the interaction between DNA and histone residues, while HDACs remove acetyl groups [157].

The vital role of the acetylation process in maintaining the balance between osteoblastic bone formation and osteoclastic bone resorption is crucial for bone tissue homeostasis [158]. The acetylation of H3K14 (histone H3, lysine 14) and H3K9 (histone H3, lysine 9) can promote osteogenic differentiation of dental-derived MSCs [159]. HDACs are downregulated during the osteogenic differentiation of dental-derived MSCs [160, 161]. The use of HDAC inhibitors effectively increases the acetylation of H3K9K14 (histone H3, lysines 9 and 14) and promotes the expression of bone-related genes [162, 163]. Valproic acid (VPA), a short-chain fatty acid, can inhibit class II HDACs. Paino et al. demonstrated that HDAC2 silencing in DPSCs leads to increased expression of OPN and bone sialoprotein (BSP) and downregulates the mRNA levels of osteocalcin (OCN), which resembles the effect of VPA [164]. This result suggests that the specific inhibition of an individual HDAC by RNA interference could only enhance a single aspect of osteoblast differentiation, resulting in selective effects. It has been reported that the glucocorticoid receptor (GR) plays a key role in this regulation. HDAC2 binds to GR and inhibits its translocation into the nucleus; however, when HDAC2 is inhibited by VPA, GR can enter the nucleus, thereby affecting the expression of OCN [165].

Histone deacetylase 6 (HDAC6) is a class IIb HDAC with a unique duplicated deacetylase domain and ubiquitin-binding domain [166]. Interestingly, HDAC6, as a critical regulator of PDLSCs aging, can deacetylate p27Kip1. Loss-of-function experiments suggested that pharmacologic inhibition of the deacetylase activity of HDAC6 accelerated PDLSCs senescence and impaired MSCs activities, which showed reduced osteogenic differentiation and diminished migration capacities; thus, HDAC6 may be a new target for intervention in the aging process of PDLSCs [167].

### 4.3. Noncoding RNAs

Noncoding RNAs (ncRNAs) play an essential role in histone modification, gene silencing, and targeting DNA methylation. They are divided into short ncRNAs, with lengths of less than 30 nucleotides, and long ncRNAs, with more than 200 nucleotides [168]. ncRNAs regulate gene expression through transcription and posttranscriptional control. The overall activity and functional balance of gene networks are maintained by lncRNA/miRNA/mRNA regulatory interactions [169]. For example, a total of 89 lncRNAs, 1,636 mRNAs, and 113 miRNAs were differentially expressed after DPSCs differentiation. Simultaneously, an array of signaling pathways, including phosphoinositide-3-kinase–protein kinase B, TGF-*β*, and Wnt, were also affected. The lncRNA SNHG7 was shown to inhibit the odonto/osteogenic differentiation of DPSCs when silenced [170].

MicroRNAs (miRNAs) are vital regulators that promote the intrinsic properties of MSCs, such as their self-renewal, pluripotency, and differentiation capacities, and miRNAs have a length of approximately 20-22 nucleotides. MiRNAs extensively regulate cell functions by affecting the abundance and translation efficiency of homologous mRNAs. Single miRNAs can target numerous gene sites on mRNA transcripts. In contrast, targeting multiple miRNAs can jointly target a single mRNA [171–173]. MiRNAs are believed to be novel regulators in the differentiation of dental MSCs by targeting related genes. During osteogenic differentiation, the expression of 116 miRNAs was altered significantly in PDLSCs. The upregulated miRNAs were miR-654-3p and miR-4288 and the downregulated miRNAs were miR-34c-5p, miR-218-5p, miR-663a, and miR-874-3p. The prediction of target genes suggested that these significantly altered miRNAs may impact the osteogenic differentiation of PDLSCs by targeting osteogenesis-related genes [174].

Long noncoding RNAs (lncRNAs) (>200 nucleotides) are the largest ncRNA transcript family in the human genome and participate in transcription and posttranscriptional and epigenetic regulation of genes [175]. lncRNAs can fold into complex secondary or higher-order structures, and they show greater potential and versatility for gene regulation than miRNAs [176]. lncRNAs can act as RNA decoys and miRNA target site decoys. They bind to specific combinations of regulatory proteins and play essential roles in chromatin modification and processing of mRNA targets. A recent study showed that lncRNAs can cross-talk with mRNAs through competition for shared miRNA-response elements. In this circumstance, lncRNAs function as competitive endogenous RNAs (ceRNAs), which correspond to miRNA sponges or antagomirs, to affect the expression levels and activities of miRNAs, thereby repressing miRNA targets and causing an additional level of posttranscriptional regulation [177]. Previous studies have shown that lncRNAs regulate gene expression and function by competing with miRNAs for binding to target mRNAs [178]. Two thousand and one hundred sixty-two lncRNAs were differentially expressed between PDLSCs and GMSCs. These lncRNAs could be potential regulators, especially those with higher fold change (FC), such as lncRNA-n336841, lncRNA-n341766, and lncRNA-n333720 [179].

Circular RNAs (circRNAs) are widely distributed in organisms and represent a type of ncRNA with a cyclic covalent structure that has a high degree of evolutionary conservation and tissue cell expression specificity [180, 181]. circRNAs are more stable than linear RNAs due to their resistance to ribonuclease digestion [182]. Chen et al. revealed the circRNA expression profile in DPSCs during odontogenic differentiation. 43 upregulated circRNAs and 144 downregulated circRNAs were found in the process of dental differentiation. These differentially expressed genes are rich in signaling pathways that regulate the pluripotency of MSCs, such as the Wnt signaling pathway and the TGF signaling pathway [183]. Recently, circRNAs were also shown to function as ceRNAs to regulate the effect of miRNAs on their target genes during cell differentiation. Previous studies have found that circRNA cerebellar degeneration-related protein 1 (CDR1) competitively inhibits miR-7 and stimulates the expression of growth differentiation factor- (GDF-) 5, thereby promoting the osteogenic differentiation of PDLSCs. This process activates the Smad1/5/8 and p38 MAPK differentiation pathways [184]. In addition, CDR1as acted as a miR-7 sponge to activate the ERK signaling pathway and thus mediated the inhibitory effect of lipopolysaccharide (LPS) on cell proliferation. Knockdown of CDR1as promotes the inhibition of PDLSCs proliferation induced by LPS [185].

## 5. Conclusions

Among the regenerative strategies, dental-derived MSCs-based techniques have demonstrated particular promise [186, 187]. Several preclinical studies and clinical trials have been performed using dental-derived MSCs for the treatment of dental and nondental diseases, such as neurodegenerative diseases and autoimmune and orthopedic disorders [188–191]. Moreover, no adverse events that may be related to cell transplantation have been reported [192, 193]. These suggest the efficacy and safety of dental-derived MSCs-based therapy. However, previous studies have illustrated the difficulty in generating a consistent population of cells for therapeutic use. Even with tissue from a single donor, controlled culture conditions, and the expansion of single cells, each clone produces a distinct population with widely different morphology, growth kinetics, gene expression profile, and epigenetic status [194–196]. Based on this, we could consider that it may be necessary in the future to establish MSCs banks based on the heterogeneity of dental-derived MSCs, in case of a need to screen for cells prior to clinical use [12].

In addition, clonal cultures serve as an extremely useful research tool to identify desirable properties of cells within mixed populations. In future studies, screening of single cell-derived clones on a larger scale to that described in this report will serve to further understand cell heterogeneity and its impact on the development of MSCs-based therapies [50]. We recommend that, whenever possible, studies performed at the population level should be validated in terms of the principal findings using clonally expanded populations. This would clarify whether the response is common to all MSCs, or only to selected subpopulations [22]. Moreover, functional diversity within a MSCs colony must be considered in the design of experiments and trials for even nonclonal MSCs populations and can be mitigated or even exploited when the mechanisms of onset are better understood.

Cell therapy entails the administration of living cells that have been purified, propagated, or differentiated to create a cell product for a specific therapeutic need [197]. Identifying key markers that support cell functions is a significant aspect of the development of dental-derived MSCs therapies. It allows the optimization of population selection by selectively screening and isolating better quality dental-derived MSCs for in vitro expansion and assessment, aiding the translational development of more effective MSCs-based therapies for clinical evaluation and application [76]. Strategies to isolate, purify, and propagate subpopulations of adult MSCs may, therefore, contribute to the development of cell therapy products with enhanced clinical benefit in the future. Moreover, cell reprogramming and the induction of pluripotency depend critically on the control of the epigenetic tags linked to cell differentiation [198]. Therefore, the study of these multiple epigenetic modifications associated with the differentiation process, and how these could be reversed, is of paramount importance [25, 140, 199]. The ideal situation is when key markers of dental-derived MSCs could be analyzed and used to identify different cell types or subpopulations in the complex tissue [200]. The epigenome information from the same set of single cells could be used subsequently to investigate how different epigenetic layers regulate transcription [201]. Finally, to build a causal relationship between genotype and phenotype, it will be ideal to knock out key component genes for MSCs in vivo using gene-editing technologies [202, 203]. This control over dental-derived MSCs composition and function will accelerate the translation of cell therapy into clinical practice.

Although the results of the present research on dental-derived MSCs are promising, many of the key markers and epigenetic modifications discussed here have yet to be validated in an animal model [81, 89, 204]. There are currently less clinical research reports on dental-derived MSCs. A key challenge in therapeutic application of MSCs appears to be that the surface markers commonly related to in vitro functionality are not necessarily associated with the corresponding activity in vivo [205]. Based on this, we encourage verifying first in animal models and then in clinical trials all the promising surface markers and epigenetic modifications that have been identified based on the in vitro function of MSCs. Key molecules that are predictive of clinical outcome are candidates to use as quality attributes for robust and reproducible manufacturing of MSCs therapies [20, 206, 207]. Moreover, clinicians need to be encouraged to pay more attention to the research progress of dental-derived MSCs and develop new methods for clinical application. Small advances in the clinical application of dental-derived MSCs will bring great encouragement to researchers [208–210]. Similarly, the development of basic research will accelerate the clinical application of dental-derived MSCs [211, 212].

## Figures and Tables

**Table 1:** Characteristics of dental-derived MSCs.

Cell type	Origin	Multipotentiality (*in vitro*)	Application	References
DPSCs	Dental pulp tissue	Osteo/odontogenic	Immunoregulation	[213–217]
		Adipogenic	Angiogenesis	
		Chondrogenic	Nerve injure treatment	
		Vascular		
		Neurogenic	Dentin/pulp complex formation	

SHEDs	Human exfoliated deciduous teeth	Osteo/odontogenic	Angiogenesis	[46, 218–220]
		Adipogenic	Dental/pulp complex formation	
		Chondrogenic		
		Neurogenic		

SCAPs	Apical papilla	Osteo/odontogenic	Dentin/pulp complex formation, spinal injure treatment Angiogenesis	[221, 222]
		Adipogenic		
		Neurogenic		

PDLSCs	Periodontal ligament	Osteo/cementogenic	Immunosuppressive effects	[8, 223–225]
		Adipogenic	Periodontal disease	
		Chondrogenic		
		Neurogenic		

DFCs	Dental follicle	Osteo/cementogenic	Periodontal tissue	[54, 226, 227]
		Odontogenic	Angiogenesis	
		Adipogenic	Pulp tissue formation	
		Chondrogenic		
		Neurogenic		

TGSCs	Apical papilla in the developing tooth germ	Adipogenic	Liver disease Dental defects	[32, 228, 229]
		Osteogenic		
		Neurogenic		
		Adipogenic		
		Chondrogenic		
		Endothelial		

GMSCs	Gingiva	Osteogenic	Nerve regeneration, mandibular defects	[31, 43, 230]
		Chondrogenic		
		Neurogenic		
		Adipogenic	Immunomodulatory properties	

**Table 2:** Key markers of dental-derived MSCs.

Study mode	Characterization	Key markers	Mechanism	Function (partly)	References
*Proliferation potential*					
Human SCAPs					
(i) STRO-1^+^/CD146^+^ (ii) STRO-1^−^/CD146^+^	(i) Plastic adherent (ii) Osteo/odontogenic, adipogenic, neurogenic differentiation	STRO-1	(i) (+) DSPP, BSP, ALP, BGLAP, BMP2, Runx2, NFL-L, nestin, NCAM, and *β*-tub-III	In vitro (i) As a marker for subpopulation (ii) With higher CFU efficiency	[81]
Human PDLSCs					
(i) ALP^+^ (ii) ALP^−^	(i) Plastic adherent (ii) Osteogenic, adipogenic, and neurogenic differentiation (iii) Positive for STRO-1, CD73, CD90, CD106, and CD146	ALP	(i) (+) STRO-1, CD146 NANOG, OCT4, SOX	In vitro (i) As a marker for subpopulation (ii) Express higher level of stemness genes	[89]
DFCs (rat)					
(i) Overexpress NFIC	(i) Plastic adherent (ii) Colony formation (iii) Osteogenic and adipogenic differentiation (iv) Positive for CD29, CD44, CD90, and negative for CD34 and CD45	NFIC	(i) (+) ALP, Col I, Runx2	In vitro (i) As a marker for regulating DFCs (ii) Promote the proliferation and osteogenic/cementogenic differentiation	[91]
*Differentiation potential*					
Human DPSCs		CD146			
(i) CD146+ (ii) CD146- (iii) CD146+/-	(i) Plastic adherent (ii) Osteogenic and adipogenic differentiation		(i) (+) ALP, osteocalcin, DMP1, DSPP	In vitro (i) As a marker for subpopulation (ii) With higher osteo/dentinogenic and adipogenic differentiation In vivo (i) Generated clear dentin/pulp-like structures	[98]
Human DPSCs		CD271/ p75 NTR,			
(i) CD271^+^ (ii) CD271^−^	(i) Plastic adherent		(i) (+) nestin, SOX1, SOX2 and SOX9	In vitro (i) As a marker for subpopulation (ii) With greater neural differentiation potential	[102]
Human DPSCs (i) STRO-1^+^/c-Kit^+^/CD34^+^ (ii) STRO-1^+^/c-Kit^+^/CD34^−^	(i) Plastic adherent (ii) Osteogenic and adipogenic differentiation	CD34	(i) (+) CD271 and nestin, *β*-galactosidase, MAP-2, Neu-N, synapsin	In vitro (i) As a marker for subpopulation (ii) With higher efficiency of neurogenic commitment	[106]
Human SCAPs		WIF1			
(i) Overexpress WIF1	(i) Plastic adherent		(i) By activating OSX (ii) (+) ALP, DSPP, and DMP1	In vitro (i) As a marker for regulating SCAPs (ii) Enhance dentinogenic differentiation potential In vivo (i) Generated greater bone/dentin-like tissues	[108]
Human SCAPs		GNAI3			
(i) Knockdown GNAI3	(i) Plastic adherent (ii) Odonto/osteogenic differentiation (iii) Positive for CD44 and CD90 but negative for CD14 and CD45		(i) By suppressing JNK/ERK signaling (ii) (+) DSPP, Runx2, OSX, OPN, OCN, and BMP4	In vitro (i) As a marker for regulating SCAPs (ii) Promoting proliferation, migration and odonto/osteogenic differentiation	[117]
*Immunomodulatory*					
Human PDLSCs					
(i) STRO-1^+^/CD146^+^ (97.1%)	(i) Plastic adherent	STRO-1/CD146	(i) (-) CD1b	In vitro (i) As a combination of markers for subpopulation (ii) Regulating DC-mediated T-cell proliferation	[124]
*Cellular aging*					
Human DPSCs					
(i) Young DPSCs/MDPSCs (ii) Aged DPSCs/MDPSCs	(i) Plastic adherent (ii) Angiogenic, neurogenic, odontogenic/osteogenic, and adipogenic differentiation (iii) Positive for CD29, CD44, CD73, and CD90, and negative for CD31	Migratory response to G-CSF	(i) A small age-dependent increase: SA-*β*-gal, p16, p21, IL-1*β*, IL-6, IL-8, Gro*α*	In vitro (i) As a marker for subpopulation (ii) With high proliferation, migration, and regeneration potential is independent of age	[130]
Human PDLSCs					
(i) Activate YAP	(i) Plastic adherent (ii) Osteogenic, chondrogenic, and adipogenic differentiation (iii) Positive for CD44, CD73, CD90, and CD105), and negative for CD11b, CD19, CD34, CD45, HLA-DR	YAP	(i) (+) P-MEK, P-ERK, P-P90RSK and P-Msk (ii) (-) Bcl-2 family members (Bak, Bid, and Bik)	In vitro (i) As a marker for regulating PDLSCs (ii) Promote proliferation, accelerating the cell cycle, inhibiting apoptosis, and delaying senescence	[135, 136]
Human DPSCs					
(i) Overexpress TGF-*β*1	(i) Plastic adherent (ii) Osteogenic, chondrogenic and adipogenic differentiation (iii) Positive for CD90, CD44, CD105, and CD73, and negative for CD34, CD45, CD11b, and HLA-DR	TGF-*β*1		In vitro (i) As a marker for regulating DPSCs (ii) Have positive effect on proliferation, cell cycle, and prevents cellular senescence and apoptosis	[40]

**Table 3:** Epigenetic modifications of dental-derived MSCs.

Study mode	Characterization	Epigenetic molecules	Mechanism	Function (partly)	References
Human DPSCs/peri-implantitis DPSCs					
(i) Upregulate LINC00968	(i) Plastic adherent (ii) Osteogenic differentiation	LINC00968	(i) LINC00968 regulates RUNX2 expression by sponging miR-3658 (ii) (+) Runx2, Osx, and ALP	In vitro (i) As a therapeutic target of DPSCs (ii) Promote osteogenic differentiation and bone formation In vivo (i) Generate new bone and nodes in graft	[178]
Human DPSCs					
(i) Overexpress lncRNA-CCAT1	(i) Plastic adherent	lncRNA-CCAT1	(i) (+) collagen I, OPN, and OCN (ii) (-) miR-218	In vitro (i) As a therapeutic target of DPSCs (ii) Promote cell proliferation and differentiation	[231]
Human DPSCs					
(i) Overexpress lncRNA H19	(i) Plastic adherent (ii) Osteogenic differentiation (iii) Positive for CD73, CD90, CD105, and CD146, and negative for CD34 and CD45	lncRNA H19	(i) By inhibiting the DNMT3B-mediated methylation of DLX3 (ii) (-) SAHH	In vitro (i) As a therapeutic target of DPSCs (ii) Promote the odontogenic differentiation	[204]
Human DPSCs					
(i) Overexpress lncRNA MEG3	(i) Plastic adherent (ii) Osteogenic differentiation	lncRNA MEG3	(i) (+) SMURF1 (ii) (-) miRNA-543, OSX, OPN, OCN, and RUNX2	In vitro (i) As a therapeutic target of DPSCs (ii) Inhibit osteogenic differentiation	[232]
Human DPSCs					
(i) Silence SNHG7	(i) Positive for CD29, CD44, and CD90 (ii) Osteogenic differentiation	lncRNA SNHG7	(i) (+) miR-1226-3p and miR-210-5p (ii) (-) OCN, ALP, DMP1, BSP, BMP2, and DSPP	In vitro (i) As a therapeutic target of DPSCs (ii) Inhibits osteogenic differentiation	[170]
Human PDLSCs					
(i) Inhibit HDAC6	(i) Plastic adherent (ii) Osteogenic differentiation (iii) Positive for CD73, CD90, CD146, and CD29 and negative for CD34 and CD45	HDAC6	(i) (-) Acetylation of p27Kip1	In vitro (i) As a therapeutic target of PDLSCs (ii) Accelerate senescence, reduced osteogenic differentiation, diminished migration capacities	[167]
Human PDLSCs					
(i) Knockdown circRNA CDR1as	(i) Plastic adherent (ii) Osteogenic differentiation	circRNA CDR1as	(i) (+) miR-7 (ii) (-) GDF5(BMP14), Smad1/5/8, and p38 MAPK phosphorylation	In vitro (i) As a therapeutic target of PDLSCs (ii) Inhibit osteogenic differentiation (iii) In vivo (iv) Less bone formation and a larger defect area	[184]
Human PDLSCs					
(i) Overexpress miRNA-132	(i) Plastic adherent (ii) Osteogenic and adipogenic differentiation (iii) Positive for STRO-1, CD73, CD90, CD105, and CD146 and negative for CD14, CD19, CD34, CD45, and HLA-DR	miR-132	(i) (-) GDF5, Runx2, OCN, and ALP	In vitro (i) As a therapeutic target of PDLSCs (ii) Inhibits osteogenic differentiation	[233]
Human PDLSCs					
(i) Downregulate lncRNA MEG3	(i) Plastic adherent (ii) Osteogenic differentiation (iii) Positive for CD90, CD105, and CD146 and negative for CD45, CD34, CD11b, CD19, and STRO-1	lncRNA MEG3	(i) (+) IGF1 (ii) (-) miRNA-27a-3p	In vitro (i) As a therapeutic target of PDLSCs (ii) Promote osteogenic differentiation	[234]
Human PDLSCs					
(i) Overexpress lncRNA SNHG1	(i) Plastic adherent (ii) Osteogenic, chondrogenic, and adipogenic differentiation (iii) Positive for CD73 and CD90 and negative for CD31 and CD34	lncRNA SNHG1	(i) By EZH2-mediated H3K27me3 methylation of KLF2 promotor (ii) (-) OCN, OSX, and ALP	In vitro (i) As a therapeutic target of PDLSCs (ii) Inhibit osteogenic differentiation	[235]
PDLSCs from periodontitis patients					
(i) Overexpress lncRNA -POIR	(i) Plastic adherent (ii) Osteogenic differentiation	lncRNA-POIR	(i) (+) FoxO1 (ii) (-) miR-182, cyclin D1, c-myc, and Axin	In vitro (i) As a therapeutic target of PDLSCs (ii) Promote osteogenic differentiation (iii) In vivo (iv) Promote osteogenesis	[236]
Human PDLSCs					
(i) Upregulate lncRNA TUG1	(i) Plastic adherent (ii) Osteogenic differentiation (iii) Positive for STRO-1 and CD146 and negative for CD45 and CD31	lncRNA TUG1	(i) (+) Lin28A, Runx2, OCN, and ALP	In vitro (i) As a therapeutic target of PDLSCs (ii) Promote osteogenic differentiation	[237]
Human PDLSCs					
(i) Overexpress lncRNA TUG1	(i) Plastic adherent (ii) Osteogenic differentiation (iii) Positive for STRO-1 and CD146 and negative for CD34 and CD45	lncRNA TUG1	(i) (+) Smad2/7, Runx2, OCN, and ALP (ii) (-) miRNA-222-3p	In vitro (i) As a therapeutic target of PDLSCs (ii) Promote osteogenic differentiation	[238]
Human PDLSCs					
(i) Silence lncRNA XIST	(i) Plastic adherent (ii) Osteogenic differentiation	lncRNA XIST	(i) (+) Runx2, OCN, and ALP (ii) (-) miR-214-3p	In vitro (i) As a therapeutic target of PDLSCs (ii) Promote osteogenic differentiation	[239]
Human PDLSCs					
(i) Overexpress lncRNA MORT	(i) Plastic adherent	lncRNA MORT	—	In vitro (i) As a therapeutic target of PDLSCs (ii) Inhibit proliferation, affect the recurrence of periodontitis	[240]
Human SCAPs					
(i) Depletion of KDM2A or BCOR	(i) Plastic adherent (ii) Osteogenic differentiation	KDM2A/BCOR (Complex)	(i) By promoting methylation of the SFRP2 (ii) (+) OSX	In vitro (i) As a therapeutic target of SCAPs (ii) Promote osteo-/dentinogenic differentiation In vivo (i) Enhance bone/dentin-like tissue formation	[112]
Human SCAPs					
(i) Silence KDM2A	(i) Plastic adherent (ii) Chondrogenic and adipogenic differentiation	KDM2A	(i) (+) SOX2 and NANOG	In vitro (i) As a therapeutic target of SCAPs (ii) Enhance adipogenic and chondrogenic differentiation	[39]
Human SCAPs					
(i) Overexpress DLX5	(i) Plastic adherent (ii) Osteogenic differentiation	DLX5	(i) (+) KDM4B, DSPP, DMP1, OPN, and OSX	In vitro (i) As a therapeutic target of SCAPs (ii) Promote osteo-/dentinogenic differentiation In vivo (i) Promote osteo-/dentinogenesis	[241]
Human SCAPs					
(i) Overexpress KDM2B	(i) Plastic adherent (ii) Chondrogenic differentiation	KDM2B	(i) (-) COL1, COL2, and SOX9	In vitro (i) As a therapeutic target of SCAPs (ii) Inhibit the chondrogenic differentiation	[242]
Human SCAPs					
(i) Knock down KDM3B	(i) Plastic adherent (ii) Osteogenic differentiation	KDM3B	(i) (-) ALP, RUNX2, OSX, DSPP, and OCN	In vitro (i) As a therapeutic target of SCAPs (ii) Inhibit the osteo-/odontogenic differentiation, proliferation, and migration	[243]
Human SCAPs					
(i) Overexpress miR-34a	(i) Plastic adherent (ii) Osteogenic differentiation	miR-34a	(i) (-) NOTCH2 (ii) (+) DSPP, RUNX2, OSX, and OCN	In vitro (i) As a therapeutic target of SCAPs (ii) Promote osteo-/odontogenic differentiation	[244]
Human SCAPs					
(i) Overexpress miR-141-3p	(i) Plastic adherent (ii) Positive for CD29, CD73, CD90, and CD105 and negative for CD34 and CD45	miR-141-3p	(i) (-) YAP	In vitro (i) As a therapeutic target of SCAPs (ii) Imped proliferative ability, promote senescence	[245]
Human SCAPs					
(i) Overexpress lncRNA-H19	(i) Plastic adherent (ii) Osteogenic differentiation	lncRNA-H19	(i) (+) miR-141, SPAG9 (activate p38 and JNK pathways), Runx2, DSP, and ALP	In vitro (i) As a therapeutic target of SCAPs (ii) Promote osteo/odontogenic In vivo (i) Enhance the osteo/dentinogenesis	[246]
Human DFCs					
(i) Downregulate lncRNA MEG3	(i) Plastic adherent (ii) Osteogenic differentiation (iii) Positive for CD29, CD44, and CD90 and negative for CD31, CD34, and CD45	lncRNA MEG3	(i) Activate Wnt/*β*-catenin signaling pathway by decreasing H3K27me3 occupation at the Wnt gene promoters (ii) (+) *β*-catenin, ALP, RUNX2, and OCN (iii) (-) EZH2	In vitro (i) As a therapeutic target of DFCs (ii) Promote osteogenic differentiation	[247]
Human DFCs					
(i) Overexpress lncRNA HOTAIRM1	(i) Plastic adherent (ii) Osteogenic differentiation	lncRNA HOTAIRM1	(i) By maintaining the hypomethylated state of the HOXA2 promoter (ii) (+) HOXA2, ALP, and RUNX2 (iii) (-) DNMT1	In vitro (i) As a therapeutic target of DFCs (ii) Inhibit proliferation and promote osteogenesis	[248]
Human DFCs					
(i) Overexpress miR-101	(i) Plastic adherent (ii) Osteogenic differentiation	miR-101	(i) (+) SP7 (osterix)	In vitro (i) As a therapeutic target of DFCs (ii) Promote osteogenic differentiation	[249]
Human GMSCs					
(i) Overexpress miR-3940-5p	(i) Plastic adherent (ii) Osteogenic differentiation	miR-3940-5p	(i) (+) p15INK4b, p18INK4c, p19INK4d, Cyclin A, DSPP, DMP1, and DLX 5 (ii) (-) Cyclin E	In vitro (i) As a therapeutic target of GMSCs (ii) Inhibit cell proliferation, enhanced the osteo/dentino genic differentiation	[250]

## Abbreviations

MSCs: Mesenchymal stromal cells
SCs: Stromal cells
BMMSCs: Bone marrow mesenchymal stromal cells
DPSCs: Mesenchymal stromal cells of dental pulp
SCAPs: Mesenchymal stromal cells of apical papilla
SHEDs: Mesenchymal stromal cells of human exfoliated deciduous teeth
PDLSCs: Mesenchymal stromal cells of periodontal ligament
DFCs: Dental follicle cells
GMSCs: Mesenchymal stromal cells of gingiva
TGSCs: Mesenchymal stromal cells of human tooth germ
FASL: Fas-ligand
N-GMSCs: Cranial neural crest cell-derived GMSCs
M-GMSCs: Mesoderm GMSCs
YAP1: Yes-associated protein 1
PD: Population doublings
Map2: Microtubule-associated protein 2
NF: Neurofilament
OCT-4: Octamer-binding transcription factor-4
SSEA-3: Stage-specific embryonic antigen-3
iPS: Pluripotent stromal
ES: Embryonic stromal
PGCs: Primordial germ cells
ALP: Alkaline phosphatase
SOX2: Sex-determining region Y-box-2
NFIC: Nuclear factor I-C
DMP1: Dentin matrix protein-1
DSPP: Dentin sialophosphoprotein
NTR: Neurotrophin receptor
SVZ: Subventricular zone
SGZ: Subgranular zone
DLX5: Distal-less homeobox 5
BGLAP: Bone gamma-carboxyglutamate protein
GFAP: Glial fibrillary acidic protein
S100B: S100 calcium binding protein B
WIF1: Wnt inhibitory factor 1
SFRPs: Secreted frizzled-related proteins
GNAIs: Guanine nucleotide binding proteins
HERS: Hertwig's epithelial root sheath
JNK: c-Jun N-terminal kinase
ERK: Extracellular-signal regulated kinase
IFN-*γ*: Interferon-gamma
PBMC: Peripheral blood mononuclear cell
G-CSF: Granulocyte colony stimulating factor
MDPSCs: DPSC subsets based on their migratory response to G-CSF
SA-*β*-gal: Senescence-associated *β*-galactosidase
CFU: Colony-forming unit
OCT-4: Octamer-binding transcription factor-4
IL-1*β*: Interleukin-1*β*
TGF-*β*1: Transforming growth factor-*β*1
ROS: Reactive oxygen species
PMDs: Partially methylated domains
ICM: Inner cell mass
DNMTs: DNA methyltransferases
5-Aza: 5-Azacytidine
5-Aza-dC: 5-Aza-2′-deoxycytidine
5-aza-CdR: 5-Aza-2′-deoxycytidine
OPN: Osteopontin
Pax7: Paired box 7
Myod1: Myogenic differentiation 1
Tet: Ten-eleven translocation
5-Mc: 5-Methylcytosine
5-hmC: 5-Hydroxymethylcytosine
DKK-1: Dickkopf WNT signaling pathway inhibitor 1
HATs: Histone acetyltransferases
HDACs: Histone deacetylases
H3K14: Histone H3, lysine 14
H3K9: Histone H3, lysine 9
H3K9K14: Histone H3, lysines 9 and 14
VPA: Valproic acid
BSP: Bone sialoprotein
OCN: Osteocalcin
GR: Glucocorticoid receptor
HDAC6: Histone deacetylase 6
ncRNAs: Noncoding RNAs
miRNAs: MicroRNAs
lncRNAs: Long noncoding RNAs
ceRNAs: Competitive endogenous RNAs
circRNAs: Circular RNAs
FC: Fold change
CDR1: Cerebellar degeneration-related protein 1
GDF: Growth differentiation factor
SFRPs: Secreted frizzled-related proteins.
